# Organizing Psycho-Oncological Care for Cancer Patients: The Patient’s Perspective

**DOI:** 10.3389/fpsyg.2021.625117

**Published:** 2021-04-22

**Authors:** Anouk S. Schuit, Karen Holtmaat, Valesca van Zwieten, Eline J. Aukema, Lotte Gransier, Pim Cuijpers, Irma M. Verdonck-de Leeuw

**Affiliations:** ^1^Department of Clinical, Neuro and Developmental Psychology, Faculty of Behavioral and Movement Sciences, Amsterdam Public Health Research Institute, Vrije Universiteit Amsterdam, Amsterdam, Netherlands; ^2^Cancer Center Amsterdam (CCA), Amsterdam UMC, Vrije Universiteit Amsterdam, Amsterdam, Netherlands; ^3^Amsterdam UMC, Otolaryngology—Head and Neck Surgery, Cancer Center Amsterdam, Vrije Universiteit Amsterdam, Amsterdam, Netherlands; ^4^Ingeborg Douwes Centrum, Center for Psycho-Oncology, Amsterdam, Netherlands; ^5^Department of Psychiatry and Medical Psychology, OLVG Hospital, Amsterdam, Netherlands

**Keywords:** patient preferences, supportive care, psychological care, psycho-oncological care, cancer distress

## Abstract

**Background:**

Cancer patients often suffer from psychological distress during or after cancer treatment, but the use of psycho-oncological care among cancer patients is limited. One of the reasons might be that the way psycho-oncological care is organized, does not fit patients’ preferences. This study aimed to obtain detailed insight into cancer patients’ preferences regarding the organization of psycho-oncological care.

**Methods:**

18 semi-structured interviews were conducted among cancer patients. Patients completed psycho-oncological treatment between 2015 and 2020 at the psychology department in a general hospital or a center specialized in psychological cancer care in the Netherlands. The interview comprised questions related to preferences regarding the institute where to receive treatment, the psychologist who provides treatment, and the type of treatment, as well as questions related to experienced barriers and facilitators to receive psycho-oncological care. Interviews were digitally recorded and transcribed verbatim. Data were analyzed individually by two coders into key issues and themes.

**Results:**

Regarding the institute, easy accessibility and prompt availability of psychol-oncological care were considered important. Regarding the psychologist, most participants had a strong preference to be treated by a psychologist specialized in cancer or other somatic diseases. Individual face-to-face therapy was preferred above other types of treatment. Several barriers were mentioned to receive psycho-oncological treatment, among which poor accessibility to psycho-oncological care, lack of knowledge on the possibilities for psycho-oncological treatment, and stigma. Most frequently mentioned facilitators were being assertive to ask for help, having a good relationship with the healthcare professional, and the integration of psycho-oncological support within medical cancer care.

**Conclusion:**

From the patient’s perspective, the organization of psycho-oncological care for cancer patients should focus on easy accessibility and availability, delivered by specialized psychologists, and integration in medical cancer care. Online and group therapy are acceptable, but individual face-to-face therapy is preferred. It is warranted to increase awareness on psycho-oncological care targeting both patients and healthcare providers.

## Background

Many cancer patients suffer from psychological distress due to physical, psychological and social challenges related to their disease, negatively affecting their quality of life (Zabora et al., 2001; Mehnert et al., 2018; Marco and White, 2019). Although prevalence rates of distress in cancer patients vary among studies (Zabora et al., 2001; Herschbach et al., 2004; Hoffman et al., 2009; Götze et al., 2014; Mehnert et al., 2018), rates of up to 50% have been reported (Mehnert et al., 2018) and many patients need psycho-oncological care (PC) (Smith et al., 2015; Wang et al., 2018). Research has shown that cancer patients’ need for PC should be an important aspect of cancer care (Brix et al., 2008; Merckaert et al., 2010; Faller et al., 2016). PC aims to improve patients’ quality of life by promoting their well-being and decreasing psychological distress (Jacobsen and Wagner, 2012). Previous studies have shown beneficial effects of PC on distress and quality of life (Rehse and Pukrop, 2003; Osborn et al., 2006; Linden and Girgis, 2012; Faller et al., 2013; Kalter et al., 2018). However, it is also known that cancer patients with psychological distress do not often use PC (Kowalski et al., 2016; Faller et al., 2017; Tondorf et al., 2018). One of the reasons may be that the way PC is organized does not fit patients’ preferences. In this paper PC is defined as professional psycho-oncological care for cancer patients provided by health psychologists by means of counseling and/or psychotherapy.

Previous studies investigating preferences regarding PC were often conducted among people with psychological problems in the general population. Preferences can relate to different aspects of psychological treatment, such as activity preferences (e.g., desires about the treatment type; individual, couple or family, or group), therapist preferences (e.g., therapist’s personality characteristics) and treatment preferences (e.g., psychotherapy or self-help interventions) (Swift et al., 2018). Complying to these preferences leads to better adherence and outcomes due to stronger therapeutic alliance, and enhanced patient-provider communication (Swift and Callahan, 2009; Swift et al., 2013, 2018; Lindhiem et al., 2014).

Integrating PC into cancer care services comes with challenges on patient, provider and institutional level (Fann et al., 2012). Earlier research among cancer patients focused primarily on their need for PC (Brix et al., 2008; Smith et al., 2015; Faller et al., 2016; Wang et al., 2018), but less on the organization of PC from patients’ perspective; why they prefer certain types of care and what facilitates or hinders the access to PC according to patients on patient, provider, and institutional level. Reasons for not using PC, on patient level, are that many patients prefer to manage their symptoms on their own and believe that their distress is not severe enough to receive PC (Mosher et al., 2014; Clover et al., 2015). Dealing with symptoms on your own could be a good strategy, since stepped care (including watchful waiting and self-care as first steps) was found to be (cost)effective (Krebber et al., 2016; Jansen et al., 2017). In stepped care, patients not benefitting from self-care and low-intensity interventions are offered to “step-up” to professional PC. However, on provider level, and outside a stepped care context, a lack of knowledge of PC among both patients and physicians is found to be a barrier to receive PC (Mosher et al., 2010, 2014; Dilworth et al., 2014). On institutional level, it may be difficult for patients to find their way within the complex healthcare system and to know where to find PC. In the Netherlands, PC for cancer patients can be provided at different locations; in the hospital (e.g., by psychologists at the Department of Psychology), primary care (e.g., by mental health workers), or specialized centers for psychological cancer care (e.g., by psychologists) (Integraal Kankercentrum Nederland, 2017). PC can also be provided as different types: face-to-face, individually, remotely (e.g., online therapy), together with a partner or other relatives, in a group, or blended (i.e., blending different types of care) (Jaspers and Van Middendorp, 2010). In the Netherlands, the maximum acceptable waiting time for PC is 14 weeks (GGZ Nederland, 2018). PC is often reimbursed by health insurance companies (Rijksoverheid, 2021).

Since the number of cancer patients is increasing annually (IKNL, 2014), a growing need for PC is expected in the coming years (Zegers et al., 2010). Better knowledge is needed on cancer patients’ preferences regarding the organization of PC. This will enable patient-centered care, which emphasizes individual patient preferences, needs and values (Epstein and Peters, 2009; Loiselle and Brown, 2020). Therefore, the aim of this qualitative study was to gain more understanding of the preferences regarding the organization of PC among cancer patients who received PC in the past, and the barriers and facilitators they experienced to receive PC.

## Materials and Methods/Design

### Recruitment and Study Sample

Between February 2019 and June 2020, adult cancer patients were recruited who completed psycho-oncological treatment at the Department of Psychiatry and Medical Psychology in a general hospital (OLVG) or at a center specialized in psychological cancer care [Ingeborg Douwes Centrum (IDC)], both located in Amsterdam, Netherlands. Patient referrals to IDC occurred both from hospitals and primary care centers in Amsterdam and surrounding areas. Patient referrals to the Department of Psychiatry and Medical Psychology in the hospital occurred by the physician, general practitioner (GP), or nurse specialist in the hospital.

Patients were eligible if they were 18 years or older, able to communicate in Dutch, were treated for cancer (any type and stage, treatment modality, or treatment intent), and received their psycho-oncological care prior to participation in the study (either before, during, or after cancer treatment).

Patients were screened for eligibility by the psychologist who provided their psychological treatment. Reasons not to approach patients were patients being too ill, privacy reasons, the nature of the psychological symptoms (severe post-traumatic stress disorder or changed psychopathology), and having mental disabilities. Patients were selected based on completion of their psychological treatment; at first patients were selected who finished their treatment recently. Subsequently, the time frame of completion was broadened further back in time (up until data saturation had been reached). All patients received their psychological treatment up to four years prior to study participation.

Eligible patients received a letter with information on the study. Interested patients were asked to return the reply card to the researcher of the Vrije Universiteit Amsterdam (AS) to express their interest in the study. Subsequently, they were contacted by phone to give them the opportunity to ask questions and to schedule the interview with this researcher (AS).

### Interview

A semi-structured interview scheme was used, consisting of two main topics (preferences and experiences, e.g., barriers and facilitators to receive PC) with related questions (Table 1). Flexibility was allowed in the order in which questions were asked. Topics and related questions were derived from the literature and the clinical experience of the research team. After three interviews the research team discussed whether the interview scheme had to be adapted; minor changes were made in the formulation of some sub questions. Participants were asked to provide information about their cancer diagnosis, psycho-oncological treatment and sociodemographic characteristics at the start of the interview.

**TABLE 1:** Interview topics.

Topics	Key questions
Preferences	- What are your preferences regarding the setting of care (e.g., location and type of psychologist)?
	- What are your preferences regarding the type of professional support (face to face/group sessions/online therapy)?
	- What were other preferences regarding the psychological care you wanted to receive?
Experiences	- Which barriers did you experience or what could be barriers when looking for psychological support?
	- Which facilitators did you experience or what could facilitate receiving psychological support?

Interviews were conducted by a PhD-student (AS) trained in qualitative research methods. No relationship between the interviewer and the participants was established prior to study commencement. Participants were interviewed at the location of their preference; e.g., at their homes, their workplace, or at the Vrije Universiteit Amsterdam. This study was conducted partly during the lockdown in the Netherlands (March–May 2020) due to the COVID-19 pandemic; due to safety procedures concerning COVID-19, three interviews were conducted by phone. All interviews were recorded with an audio device and transcribed verbatim. Transcripts were not returned to the participants for comments or corrections.

### Data Analysis

Data was analyzed using Atlas.ti (version 8). The transcripts of the interviews were analyzed by two coders independently (AS and VvZ), using thematic analysis (Braun and Clarke, 2006). Data-analysis ran parallel to data collection. First, the coders read the transcripts to get familiar with the data. Then, two coders analyzed the data individually, coding citations into key issues and themes, derived from the data. Findings were discussed in consensus meetings in which differences were resolved and a thematic framework was created. Two independent persons (IVdL and KH) were involved for advice, when there were doubts during the consensus meetings. All quotes extracted from the interviews, provided in this paper, were translated from Dutch into English. Information in quotes that could lead to a person’s identification was removed to ensure respondents’ privacy.

In this paper the consolidated criteria for reporting qualitative research (COREQ) were followed to report about the study (Tong et al., 2007). The study was approved by the Medical Ethical Committee of OLVG Hospital, Amsterdam (18.178 PPPSC). All participants provided written informed consent before the start of the interview.

## Results

### Study Population

In total, 85 patients were invited to participate: 26 by OLVG (31%) and 59 by IDC (69%), of whom 67 (79%) were not willing to participate [not willing to talk about the disease (*n* = 2), being too ill to participate (*n* = 1), no reason provided (*n* = 64)]. A total of 18 patients were interviewed (7 patients via OLVG, 11 patients via IDC), after which no additional information of value was obtained and data saturation had been reached. The duration of the interviews lasted 44–136 minutes (median 64). The majority of the participants was female (72%) and received PC at IDC (61%). The mean age of participants was 47 years (SD 13.1). Table 2 shows an overview of the participant characteristics.

**TABLE 2:** Participant characteristics (*n* = 18).

	n (%)
**Sex**	
Male	5 (28)
Female	13 (72)
**Age at interview (in years)**	
Mean (SD)	47 (13.1)
Minimum	24
Maximum	64
**Marital status**	
Single	1 (6)
Having a relationship/Living together	6 (33)
Married	8 (44)
Widow(er)	1 (6)
Divorced	2 (11)
**Children**	
Yes	10 (56)
No	8 (44)
**Highest level of education completed**	
Academic education	9 (50)
Higher education	7 (39)
Secondary education	2 (11)
**Current employment**	
Paid job	14 (78)
No paid job	4 (22)
**Received psychological treatment in**	
Psychology department within hospital (OLVG)	7 (39)
Psychological cancer care center (IDC)	11 (61)
Cancer diagnosis*	
Breast cancer	9 (50)
Colorectal cancer	3 (17)
Head and neck cancer	2 (11)
Hematological cancer	4 (22)
Unknown	1 (6)
**Time since cancer diagnosis**	
1–3 years	11 (61)
3–5 years	5 (28)
>5 years	2 (11)
**Time since psychological treatment**	
<1 year	4 (22)
1–3 years	11 (61)
3–5 years	3 (17)

*One participant was diagnosed with both breast cancer and colorectal cancer. Therefore, this total percentage does not add up to 100.

Most participants who received PC at OLVG or at IDC had finished their cancer treatment but still had regular follow-up sessions with their physician. Some were still undergoing cancer treatment during PC.

### Preferences

Participants described three categories related to their preferences regarding the organization of PC: the institute, the psychologist (Table 3), and the type of PC (Table 4).

**TABLE 3:** Preferences regarding the institute and psychologist.

Key issues	Themes
**Preferences related to**	
Institute	Short term availability of PC
	Accessibility:
	- Short traveling time to location
	- (Free) car parking facilities
	- Prefer to receive medical and psychological care at same location
	Institution is specialized in PC:
	- Curiosity about what a specialized center has to offer
	- Easier to fit in; everyone has cancer
	Personal feelings and experiences:
	- Feeling comfortable at the location where to receive PC
	- Experiences during medical cancer treatment (when receiving PC in the hospital)
Psychologist	Professional distance to the psychologist:
	- Easier to explain difficult topics
	- Easier to show emotions
	- Psychologist is able to put things into other perspectives
	Experienced in cancer/other physical diseases:
	- Psychologist must have knowledge about the psychological impact of diseases (e.g., cancer), the healthcare environment and about psychological mechanisms
	∘ Not having to explain things which are self-evident when having a serious illness (e.g., cancer)
	Gender:
	- Same gender due to gender related physical symptoms
	Age:
	- Being the same age category could make it easier to feel connected to the psychologist
	Professional with lots of work experience
	Good relationship with psychologist

**TABLE 4:** Advantages and disadvantages per type of care.

	Advantages	Disadvantages
Individual PC	- One-on-one setting with psychologist (having undivided attention of the psychologist)	NM
	- Possible to bring relatives to therapy	
PC in groups	- Talking to people who understand your situation and have similar problems	- Too burdensome to hear about other patients’ problems
	- Sharing experiences and advice how to cope with cancer related symptoms	- Makes you conscious that you have (had) cancer
	- Learning to accept confronting circumstances	- Feeling disappointed when peers drop-out
		- Difficult to connect with peers with different age
		- Having the feeling that it is not relevant for other peers to listen to your experiences
		- Having concerns about privacy
		- Not being able to be your true self
		- Difficult to express yourself when not feeling comfortable in a group
		- Less time available per person
Online therapy/blended therapy	- Available 24/7	- Relatively unknown area
	- Available at your own home	- Lack of social contact makes it difficult to communicate:
	- Saving traveling time	∘ Non-verbal signals and emotions are less visible
	- No waiting lists	∘ No in-depth conversations
	- Suitable for less complicated needs	∘ No direct support from psychologist
	- Extra support besides face-to-face support	- Easier to get distracted or to avoid therapy
	- Available in different languages	- Disturbing when technology does not work properly (e.g., during videoconferencing)
		- Having concerns about privacy
		- Not suitable for all patients (dyslexia, visual problems)
		- Not wanting to follow therapy in your home environment

*NM, none mentioned.

#### Preferences Regarding the Institute

The majority of the patients treated at IDC were not yet familiar with the existence of a center specialized in PC for cancer patients until their healthcare professional suggested a referral to IDC. In some cases, patients were recommended to receive PC at IDC by acquaintances (e.g., colleagues). The main reasons for participants to receive PC in the hospital instead of in a center specialized in PC for cancer patients were shorter waiting lists or practical reasons.

**Short-term availability of support** and **accessibility of the location** (e.g., short traveling time and car parking facilities) were considered important when choosing where to receive PC. Also, familiarity with the location was mentioned to prefer receiving medical and psychological care at the same location (i.e., the hospital). Some participants preferred an institute **specialized in PC**, due to curiosity or because it felt easier to fit in, because everyone treated here has or had cancer.

In addition, **personal feelings and experiences** were important; patients want to feel comfortable at the institute where they receive care and—when receiving PC in the hospital—the experiences during cancer treatment often play a major role in deciding where to receive PC:

“I felt more comfortable to be treated at IDC. Because it is a neutral environment. The hospital, that’s the place where you’ve experienced some bad things. It feels better to go to a different place.”

#### Preferences Regarding the Psychologist

Most participants preferred to receive PC by a **psychologist experienced in supporting patients with somatic diseases, or cancer in particular**. Many people indicated that it was important to receive PC from a psychologist with knowledge about the psychological impact of somatic diseases (e.g., cancer):

“Firstly, you can connect more easily [with the psychologist], because you think ‘that person understands how things in the hospital work’. Secondly, it is easier for her to give tips and tricks because she knows how the medical world works and how things work for patients who experienced stressful events in their life. I didn’t want to go to a psychologist ‘just around the corner’.”

Some participants (more often those who received PC in the hospital), indicated that they did not prefer a psychologist especially trained in treating cancer patients over a psychologist specialized in treating patients in general.

Personal factors also played a role when it came to preferences for a psychologist; wanting to be treated by a psychologist with the same (female) **gender** due to specific physical symptoms, or with the same (young) **age category** were both mentioned, such as the wish to be treated by a psychologist with **a lot of work experience** (which was not further specified).

Participants described the added value of visiting a psychologist. They appreciated the **professional distance to their psychologist**, making it easier to discuss difficult topics than with family or friends. People said it was easier to show their emotions to a psychologist than to relatives:

*“It is very difficult to see people feeling sad about you. Especially the people very close to you. [*…*] When I felt bad, I said I was feeling fine. But to [name psychologist] I could just tell ‘Well, I’m doing very badly’. Not that she doesn’t care, but she is just not personally affected.”*

“Over there [with the psychologist] I can be scared, I can also admit that I’m scared and that I’m afraid to die, and that I feel sad. But to your friends and family you always pretend to be strong, because you don’t want them to feel bad.”

Participants appreciated the psychologist helping them to see things from other points of view. They also thought it was important to have a **good relationship** with the psychologist.

#### Preferences Regarding the Type of Care

Although all participants received individual PC, everyone was asked to reflect on advantages and disadvantages of various types of PC (Table 4). Some participants also received other types of PC in their past (not always related to cancer), such as group therapy sessions.

##### Individual PC

Most participants preferred individual PC because it enabled them to talk about their problems in a one-on-one setting, while having the undivided attention of the psychologist. Additionally, being able to bring their relative(s) to their therapy sessions was appreciated.

##### PC in Groups

Participants reflected on group therapy sessions. Although most participants recognized that people might benefit from group therapy, the majority did not prefer this type of care.

Participants described that it could be pleasant to talk to people who understand your situation, because this puts things into perspective and enables you to share experiences. Furthermore, it would be helpful for learning to accept confronting circumstances (e.g., when a peer passes away during therapy).

However, people mentioned that it would be too burdensome to hear about other patients’ problems and that it could be confronting because it makes you more aware that you have (had) cancer. Being disappointed when peers drop-out of the group therapy was also mentioned not to prefer group therapy. Furthermore, having the feeling that sharing your experiences would not be useful for others and having privacy issues—not feeling safe enough to share confidential issues—were mentioned. It would also be hard to express yourself when not feeling comfortable. In addition, difficulties to connect with peers due to age differences and less time available per person were described as other disadvantages of group therapy.

##### Online Therapy

For most participants online therapy was a relatively unknown area (only one participant received PC during the COVID-19 pandemic, which made online therapy mandatory due to safety reasons). Participants reflected on different types of online therapy; guided therapy through videoconferencing or blended therapy with online and offline exercises, guided by a psychologist. They wondered how to be certain you communicate with an experienced psychologist and if the same psychologist will return every session during the therapy. They also thought online therapy would make it more difficult to communicate, because there is no direct contact with the psychologist:

*“With online therapy the psychologist cannot see the emotions. When you have a conversation, someone can see the emotions [*…*]. They can see through your eyes whether you are doing badly, or through your posture [*…*] Online, [*…*] when things are difficult in the session—I would just shut down my laptop. When you are with each other in a room, you cannot avoid it.”*

Participants indicated they would get distracted during online therapy or avoid therapy when it gets emotional or too time consuming. Furthermore, they noticed privacy concerns and thought online therapy would not be suitable for all patients (for example online exercises, when having visual problems or dyslexia). Not wanting to have therapy in your home environment and being disturbed when the technology does not work properly, were other disadvantages mentioned.

Some participants were curious about online support. Described benefits of online therapy were 24/7 availability of some forms of online therapy and availability at your own home. It could be offered directly—there are no waiting lists for this type of care—and be available in different languages to make it easier for non-natives. Participants described that it could be especially useful for patients with less complicated care needs. It was also considered useful when online therapy is available besides individual face-to-face therapy, as blended care.

### Experiences in Receiving PC

Participants mentioned several barriers and facilitators on patient, provider and institutional level, when reflecting on their experiences to receive their preferred PC (Table 5). Almost all participants expressed their satisfaction with the PC they received. Participants indicated the importance to offer tailored support. The barriers and facilitators that were mentioned related to participants’ specific experiences and their suggestions for improvement.

**TABLE 5:** Barriers and facilitators to receive PC.

Key issues	Themes	
	
	Barriers	Facilitators
**Patient level**		
Patients’ personal characteristics	- Being less assertive	- Being assertive to ask for PC
	- Feeling burdened to contact healthcare professional in hospital when having new questions about the disease and its symptoms	- Daring to be vulnerable - Allowing yourself to get PC
	- Preferring to work on mental health on your own (without support of psychologist) because you do not like to ask for help	
	- Not recognizing your need for help	
Patients’ motivation or personal reasons	- Having no earlier experience with PC (in general)	- Being aware of healthcare support network due to own profession
	- Having negative experience with PC in the past	- Having experience with PC (in general)
	- Having to explain the “cancer story”	- Wanting to use own experiences to help others
		- Wanting to be able to explain to yourself and others what happens with you mentally when having cancer
Stigma about PC	- Confronting to be labeled as a ‘depressed person’	- Pleasant that there is a place available especially for cancer patients
	- Feels like personal failure to seek for PC	
	- Going to a psychologist has negative associations in social environment	
	- Psychologist has negative image	
Medical treatment as priority	- Medical treatment is often the first priority for patients and physicians	NM
Time investment	- Not willing to give up spare time to receive PC	NM
Role of social environment	NM	- Getting stimulated by people in social environment to find PC
**Provider level**		
Relationship with healthcare professional Role of healthcare professionals	NM - Not being aware of PC options - Not asking enough questions to get the patient to the appropriate type of care	- Easier to discuss psychological symptoms with familiar healthcare professional - Having a good relationship with healthcare professional - Normalize psychosocial symptoms and talk about psychosocial impact of cancer on daily life - Formulate PC needs from patient’s perspective
	- Lack of time during consultation in the hospital makes it difficult to talk about psychosocial symptoms	- Offer tailored support to patients (e.g., more intensive support when necessary and tailored information about supportive care options)
	- Not receiving tailored information on PC options	- Increase patients’ recognition of their own symptoms
Taboos	- Certain topics are difficult to discuss with healthcare professional (e.g., sexuality issues)	NM
**Institutional level**		
Accessibility to PC	- Not knowing where to start to find PC	- A central point of contact within the hospital
	- PC is often provided by another institute than medical treatment	∘ With knowledge of the patient’s personal situation
	- Waiting lists for PC	∘ Where patients can turn to when having questions
	- Contact with healthcare professionals in hospital is less intensive when medical treatment is finished, making it more difficult to discuss psychological symptoms in between follow-up appointments	- An easily accessible contact outside working hours
	- Unawareness about reimbursement or financial issues holding patients back to receive PC	
	- Having to legitimize your need for help continuously	
	- Care process of psychologists is not tailored to the individual (e.g., general questionnaires used for the intake procedures)	
PC as an integrated part of cancer care	- Forcing someone to seek support could have opposite effect (i.e., patients	- Normalizing psychological impact of cancer diagnosis
resisting support)		- Making it easier for patients to accept PC
		- Informing patients in early stage of the cancer trajectory about available PC options
		- More attention to the need of PC (e.g., during follow-up period)
		- Implementing a voluntarily intake interview

*NM, none mentioned.

#### Barriers

On patient level, barriers related to **patients’ motivation and personal characteristics**. Having no earlier experience with PC or having negative experiences with PC in the past, and not preferring to explain “the cancer story” again, could be barriers to find or receive PC.

Some participants indicated wanting to cope with their mental health on their own first, without psychological help. Sometimes people prefer to solve their problems on their own and specialized care is not necessary (e.g., stepped care). However, participants also mentioned not liking to ask for help. It is more difficult to get to PC when you do not admit your need for support. Furthermore, not being assertive enough daring to ask for PC when you need it, could be a barrier. This also accounts when you feel burdened to contact the healthcare professional in the hospital when having new cancer related questions.

***Stigma attached to psychological support*** was described as a barrier to ask for PC due to different reasons: it is confronting to be labeled as “depressed person”, it feels like a personal failure to ask for PC, and going to a psychologist provokes negative reactions from the social environment:

“When I say I go to a psychologist, because I have important questions for myself, then people say ‘Are you confused, or do you have a burn-out?”’

Prejudices about the image of psychologists could also be a hurdle to overcome:

*“I always tried to keep myself away from psychological support. Because I think those people [psychologists] are weird, they have these difficult looks on their face, and they ask ‘What do you think?’. [*…*] I had that prejudice during the period I was sick, and also afterward.”*

It was mentioned that accurate timing of PC is important, because initially ***medical treatment was prioritized by patients and healthcare professionals***. Some participants indicated that shortly after the cancer diagnosis medical treatment was their first priority, being in a survival mode. They could not think about the psychological impact of cancer and also their physicians focused on medical treatment initially. Offering PC too early could counteract in accepting PC and minimize its benefits. A barrier related to ***time investment*** was the unwillingness to give up spare time for PC.

Barriers on provider level related to the ***role of healthcare professionals*** (physicians, nurse specialists, or GPs); healthcare professionals not being aware of PC options or not asking enough questions to refer the patient to the appropriate type of care. Furthermore, participants indicated that they had no overview in information about psychosocial supportive care options (including PC). Also, certain topics are difficult to discuss with healthcare professionals due to ***taboos on that topic*** (e.g., sexuality issues) or lack of time during consultations with the physician, which makes it difficult to talk about the psychosocial aspects of having cancer. However, the majority said that when they specifically asked their healthcare professional to get psychological support, they were referred to PC.

Lastly, barriers on institutional level to receive PC related to ***the accessibility of psychological care***. Participants did not know where to start finding PC due to the organization of healthcare in the Netherlands (e.g., arranging a referral from your GP and searching by yourself for a location to receive PC).

Furthermore, waiting lists for psychological care limited the access to PC. In addition, participants described that contact with healthcare professionals in the hospital gets less intensive when cancer treatment is finished, making it more difficult to discuss psychosocial symptoms that occur after treatment. PC being provided by another institute than the cancer treatment could also be a barrier. Furthermore, people mentioned that the access to mental healthcare is not tailored to the individual (e.g., filling in general questionnaires used for intake procedures).

Unawareness of financial reimbursement or having financial issues could also be barriers to receive PC; having no knowledge about the financial aspects of healthcare and not knowing that certain PC could be reimbursed by your healthcare insurance could hold people back from finding PC, thinking it is too expensive. Another barrier mentioned was (continuously) having to legitimize a need for PC:

“It disturbs me that—the psychology department is not the most approachable department of the hospital. I think that this could discourage potential patients. What bothers me most, is that I when wanted support for the second time—That you basically get a service check, as you get for your car, which I already passed the first time. So, every time you have a new question, you have to get interrogated again.”

#### Facilitators

On patient level, ***patients’ motivation*** and their ***personal characteristics*** could also serve as facilitators to receive PC. Wanting to use your own experiences to help others and being able to explain (to yourself and others) what happens to you mentally when having cancer, were personal motivations to find PC. Being aware of the healthcare support network due to participants’ profession and having earlier experience with psychological support also facilitates the access to PC. Being assertive, daring to be vulnerable, and allowing yourself to receive PC are personal characteristics making it easier to receive the preferred PC:

“If I didn’t mention that I wanted to go to that specific place, then he [the GP] would have said, just go to a psychologist. I don’t know if I would have been ended up at the IDC. I think that it matters if you are assertive and if you know your way within the healthcare system.”

*“They [healthcare professionals in the hospital] continuously asked, if you want support for this, you can just tell us. But I thought it wasn’t necessary [*…*] and then I thought, you know what, I will allow myself this, because it already sucks too much. It is available and maybe it can help me.”*

Furthermore, people’s ***social environment plays a major role*** to receive PC by stimulating patients to ask for PC when they need help. Also, specialized institutes for cancer patients facilitate the access to PC, with the potential to reduce ***stigma***; it is pleasant for patients to know that there is a place especially for cancer patients to receive PC.

A facilitator on provider level was the ***relationship with the healthcare professional*** (e.g., the physician or GP). Participants said it was easier to discuss psychological symptoms with a familiar healthcare professional. ***Healthcare professionals, such as the physician, nurse specialist or GP, also have a role organizing the referral to PC.*** When there is more attention to the psychosocial impact of cancer on daily life in general, it would be easier to discuss psychological issues with the healthcare professional. Furthermore, it would help when healthcare professionals formulate supportive care needs from the patient’s perspective and help patients to recognize their psychological symptoms. Raising awareness could facilitate looking for PC. Participants explained that tailored care is essential in PC, which means less intensive care (e.g., tailored information about supportive care options) when possible and more intensive care (e.g., therapy by a professional) when necessary.

Regarding facilitators on institutional level, people specifically mentioned ***easy accessibility*** as an important facilitator. It would facilitate the access to PC when there is a central point of contact within the hospital, familiar with patients’ personal situation, where they can turn to when having questions.

Participants indicated that***integration of PC in the cancer care process*** would help to get to the preferred PC. This could normalize the psychological impact of cancer and could make it easier to accept PC. It would also help to inform patients at an early stage of the cancer trajectory about PC options. In addition, implementing a voluntary intake interview and having more attention for the psychological care needs during the follow-up trajectory could also support finding PC.

## Discussion

This study investigated the organization of PC from the perspective of Dutch cancer patients who received PC in the past. Patients’ preferences regarding PC related to the institute where to receive care, the psychologist, and type of care. They recalled experiences that did or did not facilitate the access to PC, and reported their thoughts on what would facilitate or hinder access to PC, categorized on patient, provider and institutional level. Focusing on facilitators and resolving barriers—while taking into account patients’ preferences—in the organization of PC may support patients to timely get to their preferred PC. In this way, high-quality PC can be ensured, despite increasing demands.

The results of this study replicate existing findings, although our study sample targets a specific patient group. Many participants preferred individual therapy over group therapy, consistent to findings of earlier research (Shechtman and Kiezel, 2016; Arch et al., 2018); patients appreciate having undivided attention of the therapist and sometimes fear to expose themselves in front of others, to get lost in a group, or have concerns about privacy. Our study participants described that online therapy would be acceptable although face-to-face therapy was preferred, which is consistent with other studies investigating online therapy (Griffiths et al., 2006; Wallin et al., 2016). Previous studies described anonymity of Internet-based interventions being an advantage compared to formal mental health services, which are often still stigmatized (Andrade et al., 2014; Wallin et al., 2016). Perceived disadvantages of online therapy described in earlier research, such as lack of empathy and trust, absences of body language, and being unable to motivate yourself, were also described by participants in our study, as applies for advantages of online therapy such as having no waiting lists and 24/7 availability (Wallin et al., 2016). Patients who often use the Internet to improve their health are three times more likely to prefer Internet-based psychological interventions (Wallin et al., 2016). However, despite the relatively young age group in our study which in general frequently uses the Internet (CBS StatLine, 2019), most participants were unfamiliar with online therapy (most participants finished their psychological treatment before the COVID-19 pandemic). It has been reported that the COVID-19 pandemic seems to be great catalyst for implementation of online therapy, forcing patients and health professionals to get used to e-mental health (Wind et al., 2020). Furthermore, some patients mentioned that they would like a psychologist of the same gender and age, which corresponds to previous studies (Blow et al., 2008; Furnham and Swami, 2008; Landes et al., 2013), for example due to the nature of their presenting problem. However, there is no clear evidence on whether client-counselor similarity affects therapeutic outcomes (Blow et al., 2008; Furnham and Swami, 2008).

Patients experienced several barriers to receive PC at their preferred setting, of which some are related to non-fulfillment of certain preferences. Barriers to initiate and continue mental health treatment on patient level, among which lack of availability and stigma, were mentioned in the literature before (Tucker et al., 2013; Andrade et al., 2014; Clement et al., 2015; Boerema et al., 2016). In addition, some participants in our study mentioned that they initially preferred to self-manage psychological problems, a finding that was also documented by Baker-Glenn et al. (2011). Using the stepped care model might be interesting for these patients since previous studies revealed the positive impact of screening patients for distress followed by further assessment, appropriate referral and treatment, which could lead to improved patient outcomes (Merckaert et al., 2010; Carlson, 2013; Krebber et al., 2016). On provider level and consistent to our findings, lack of time of healthcare professionals to discuss psychosocial problems with their patients also has been mentioned as barrier in previous research (Dilworth et al., 2014). Furthermore, on institutional level, continuity of care is necessary to ensure easy access to mental health services (Mitchell et al., 2011), in which primary care plays an important role. In our study, patients reported that finding a location to receive PC by yourself could make it difficult to find your way to PC. In the Netherlands, cancer patients can be referred to PC by hospital care providers (e.g., physicians), but often referral is made by their GP (almost 40%) (Krul et al., 2012).

Integrating PC as a standard part of cancer care could be a solution to facilitate the access to PC for cancer patients (Berman et al., 2020), for example using the collaborative care approach which stimulates health professionals from both medical and psychological settings to provide integrated care, usually coordinated by a practice team or care manager (Fann et al., 2012). As described by our study participants, healthcare professionals could normalize the psychosocial impact of cancer on patients’ daily life and offer tailored support and information to patients to guide them to appropriate PC that matches their preferences. Communication skills training for healthcare professionals to integrate the discussion about psychological symptoms in their daily practice was already found to be effective (Holland, 2010; Dilworth et al., 2014; Moore et al., 2018). An open discussion about symptoms with the healthcare professional is important to let patients know that their psychosocial needs are important alongside with their medical needs (Holland, 2010; Dilworth et al., 2014). Integrating PC as standard part of cancer care will come with challenges (Fann et al., 2012), also because the number of cancer diagnoses is increasing annually. To reduce the pressure on psycho-oncological and medical healthcare services, the further implementation of the stepped care model could be a solution, which was found to be (cost-)effective to reduce distress and health-related quality of life and emphasized the importance of screening for distress in clinical practice (Krebber et al., 2016; Jansen et al., 2017).

This study provides detailed insight into the perspectives of cancer patients with respect to the organization of PC. Strengths of the study were that we included patients who received and completed psychological treatment and were thus experienced. A large variety in socio-demographic characteristics was noticed during the interviews, which suggests an appropriate level of diversity among the target group (i.e., patients with distress). However, some limitations could be addressed too, which suggest to interpret the results with caution. Information on patients’ socio-demographics and cancer diagnosis was self-reported (the research team had no access to medical records) and some patients received their psychological treatment four years prior to the interview, meaning that recall bias cannot be ruled out completely. Furthermore, we did not systematically collect data on participants’ psychiatric diagnosis, or type and format of psychological treatment.

This study used a self-selecting convenience sample, which affects generalizability and could cause selection bias which is inevitable with this recruitment method. It is possible that patients with psychological distress who did not receive psychological treatment or patients who were not treated by a psychologist specialized in cancer or other somatic diseases—who were not interviewed in this study—have other preferences regarding PC. Further research should examine why these patients did not receive PC. Some other aspects could also have affected the representativeness of our sample. Firstly, participants were treated in two centers within Amsterdam, The Netherlands. Therefore, the results could not be generalized to other hospitals or other countries. Secondly, although we aimed to recruit both patients treated with curative and palliative intent, all patients indicated their prognosis was good. However, patients’ preferences may change when they get sicker (Epstein and Peters, 2009). Lastly, our sample contained a relatively high percentage of woman (72%) and most participants were highly educated (89%). In addition, the mean age of our sample (47 years) was lower than the average age of the cancer population, which could be explained by the positive association between having distress and a younger age and/or being female (Zebrack and Isaacson, 2012; Weis et al., 2018). Another explanation could be that older patients generally have a higher disease burden and therefore are less motivated to participate in an interview study.

In conclusion, from the patient’s perspective, the organization of PC should focus on easy accessibility and availability, delivered by specialized psychologists, and integration in medical cancer care. Online and group therapy are acceptable, but individual face-to-face therapy is preferred. It is warranted to increase the awareness on the benefits and possibilities of PC targeting both patients and healthcare providers.

## Data Availability Statement

The datasets presented in this article are not readily available because the participants of this study did not consent to their data being shared. Requests to access the datasets should be directed to IVdL, im.verdonck@amsterdamumc.nl.

## Ethics Statement

This study involving human participants was reviewed and approved by the Medical Ethical Committee of OLVG Hospital, Amsterdam (18.178 PPPSC). The participants provided their written informed consent to participate in this study.

## Author Contributions

AS, KH, EA, and IVdL contributed to the design of the study. AS conducted the interviews for this study. AS, EA, and LG contributed to the data collection. AS and VZ conducted the analyses. AS, KH, and IVdL drafted the manuscript. All authors critically revised the manuscript, contributed to the article, and approved the submitted version.

## Conflict of Interest

IVdL reports grants from the Dutch Cancer Society–Alpe d’Huzes Foundation, the Netherlands Organization for Health Research and Development (ZonMw), the SAG Foundation—Zilveren Kruis Health Care Assurance Company, Danone Ecofund—Nutricia, the Dutch Society Head and Neck Cancer Patients—Michel Keijzer Foundation, Red-kite (distributor of eHealth tools), and Bristol-Myers Squibb. The remaining authors declare that the research was conducted in the absence of any commercial or financial relationships that could be construed as a potential conflict of interest.

## Abbreviations

COVID-19: coronavirus disease 2019
GP: general practitioner
IDC: Ingeborg Douwes Centrum (center specialized in psychological care for cancer patients)
NM: none mentioned
OLVG: OLVG hospital
PC: psycho-oncological care.
